# Impact of Pulmonary Artery Banding in Patients with Congenital Heart Disease and Pulmonary Hypertension

**DOI:** 10.31083/j.rcm2507253

**Published:** 2024-07-08

**Authors:** Han Zhang, Gang Li, Qiangqiang Li, Yansong Zuo, Qiang Wang

**Affiliations:** ^1^Pediatric Heart Center, Beijing Anzhen Hospital, Capital Medical University, 100029 Beijing, China

**Keywords:** congenital heart disease, pulmonary hypertension, pulmonary artery banding, competing risk analysis

## Abstract

**Background:**

To evaluate the effectiveness of the surgical approach in patients with congenital heart disease and pulmonary hypertension (PH).

**Methods:**

This was a retrospective clinical review of patients with congenital heart disease and PH who underwent pulmonary artery banding (PAB) at our institution between January 2013 and January 2023.

**Results:**

We identified 219 patients (53.4% males) with a median age of 7 (4.0–15.0) months and a median weight of 6.8 (5.2–9.0) kg at the time of PAB. The median hospital stay was 7.0 (5.0–10.0) days. The in-hospital mortality rate was 4.6%. The median follow-up was 33.0 (17.0–61.0) months. Survival rates were 96.9 ± 2.5% at 60 months and 92.1 ± 6.9% at 120 months post-PAB. 43.8% of patients had a de-banding procedure, and 147 (79.0%) patients received a second-stage procedure (34.7% univentricular, 65.3% biventricular). The mortality rate between stages was 4.3%. 21 (9.6%) patients reached a third-stage procedure. The overall mortality rate was 9.1%.

**Conclusions:**

PAB is an acceptable strategy for patients with congenital heart disease complicated with PH. The results and outcomes of subsequent univentricular or biventricular procedures are generally good.

## 1. Introduction

Patients with congenital heart disease complicated with pulmonary hypertension (PH) have historically had a poor prognosis without surgical intervention due to increased pulmonary blood flow [1]. In 1952, Muller and Dammann [2] introduced pulmonary artery banding (PAB) as a means to reduce excessive pulmonary blood flow in patients with various congenital heart disease. PAB serves as a dependable source of pulmonary blood flow and provides protection for the pulmonary vasculature. It is recommended that PAB be performed within the first 6 months of life [3]. Advancements in perioperative management have allowed for earlier definitive repair of complex congenital heart defects in small infants, resulting in a significant decrease in the need for PAB. PAB has been associated with a hospital mortality of 6.2% in 1–12 months old patients [4]. some patients present late and miss the optimal age for intervention. In these patients, a staged approach seems to be a safer option when dealing with complex anatomies and/or significant co-morbidities, as the risks associated with primary repair may outweigh its benefits [5]. Over the past few years, we have performed surgical interventions to treat these patients. Our study aims to evaluate the effectiveness of our surgical approach in treating these patients with congenital heart disease complicated with pulmonary hypertension.

## 2. Materials and Method

### 2.1 Patient Population

This was a retrospective clinical data review of patients with congenital heart disease and PH who underwent PAB at our institution between January 2013 and January 2023. Patients who underwent PAB and those who underwent concomitant surgical procedures were included. Patients who underwent bilateral branch PAB and left ventricular retraining were not included. The data were collected by retrospective review of medical records.

### 2.2 Definitions

PH was characterized by a pulmonary vascular resistance (PVR) of 2 Wood units (WU) or higher, and a mean pulmonary artery pressure (mPAP) exceeding 20 mmHg [6].

In-hospital mortality was defined as death occurring within 30 days of surgery or prior to discharge from the hospital.

Inter-stage mortality included deaths after hospital discharge and before the second-stage operation.

The degree of atrioventricular valve regurgitation (AVVR) was graded by echocardiography on an ordinal scale (1 = trivial or mild, 2 = moderate, 3 = severe). Grading was performed according to published guidelines [7]. We considered moderate and severe AAVR as clinically significant [8].

We divided patients based on whether they underwent a univentricular repair or biventricular repair.

We evaluated the effectiveness of PAB with ultrasound using the median peak Doppler velocity at the level of the banding both before discharge and during the last follow-up.

### 2.3 Surgery

Pulmonary artery banding was performed through a median sternotomy. During the surgery, a pressure-sensing tube was implanted in the distal pulmonary artery to allow for continuous measurement of intraoperative pulmonary artery pressure. Trusler’s formula (24 mm + 1 mm for each kg of body weight) was used for the initial assessment of the circumference of the band [9]. During surgery, the tightness of the band was deemed suitable when the mean pulmonary artery pressure was at or below 20 mmHg and the percutaneous arterial oxygen saturation (${\mathrm{SpO}}_{2}$) was at or above 80% while using a fraction of inspired oxygen between 21% and 40%.

### 2.4 Follow‑up

Time-related outcomes were collected by clinic visits and telephone contacts.

### 2.5 Statistical Analysis

The study utilized the Statistical Package for Social Sciences (SPSS) 25.0 software (SPSS Inc, Chicago, IL, USA) and GraphPad Prism 6 (GraphPad Software, Inc, La Jolla, CA, USA) for statistical analysis. Demographics, patient characteristics, and outcomes were expressed as median (interquartile range) or frequency (%), depending on the variable type. Categorical variables were tested using χ^2^, while numeric variables were evaluated using Mann–Whitney U tests. Univariate and multivariate logistic regression analyses were conducted to determine significant predictors of mortality, presented as odds ratio (OR) and 95% confidence interval (95% CI). The cutoff point was examined for sensitivity and specificity. Survival analysis was performed by Kaplan–Meier curves and the log-rank test. Multivariate models were applied using Cox proportional hazards for outcomes.

Progression to complete repair following univentricular and biventricular procedures, respectively, was analyzed with a competing risk framework. A significance level of *p* < 0.05 was considered statistically significant for all analyses.

## 3. Results

### 3.1 Patient Demographics

There were 117 males (53.4%). The median age of the patients was 7 (4.0–15.0) months, and the median body weight was 6.8 (5.2–9.0) kg. 11 patients had associated genetic syndromes including Downs (n = 10), Turners (n = 1). Ninety-three children were diagnosed with lung disease prior to PAB. Of these patients, 1.4% (3) patients require ventilator support treatment.

Cardiac malformations identified by echocardiography included: single ventricle (SV, n = 35), tricuspid atresia (TA, n = 20), double-outlet right ventricle (DORV, n = 57), transposition of the great arteries (TGA, n = 10), unbalanced atrioventricular septal defect (uAVSD, n = 3), balanced atrioventricular septal defect (bAVSD, n = 25), right ventricular hypoplasia (n = 2), multiple ventricular septal defects (VSDs, n = 59), and VSD with coarctation of the aorta (VSD with COA, n = 8). Of these patients, 25.6% (56) had moderate or severe AVVR.

### 3.2 Pulmonary Artery Banding Procedure

PAB was performed as an independent procedure in 184 patients, the other 35 patients underwent additional procedures, including patent ductus arteriosus (PDA) ligation (n = 24), atrioventricular valve repair (n = 3) and aortic arch repair (n = 8). The patients’ characteristics are summarized in Table 1.

**Table 1.** **Patient clinical, surgical and procedural characteristics**.

Variable			Univentricular repair group (n = 85)	Biventricular repair group (n = 134)	*p* value
Age (month)		7 (4.0–15.0)	13.0 (6.6–47.5)	5.3 (3.6–8.4)	0.000
Weight (kg)		6.8 (5.2–9.0)	8.0 (6.5–14.0)	5.9 (4.8–7.5)	0.000
Male (n, %)		117	52	65	0.072
Preoperative mPAP (mmHg)		38.0 (32.0–45.0)	38.0 (33.0–45.0)	38.0 (30.0–45.0)	0.592
Preoperative ${\mathrm{SpO}}_{2}$ (%)		98.0 (94.0–100.0)	94.0 (90.0–100.0)	100.0 (97.0–100.0)	0.000
Diagnosis (n)					
	Multiple VSDs	59	0	59	
	DORV	57	15	42	
	SV	35	35	0	
	TA	20	20	0	
	TGA	10	10	0	
	VSD with COA	8	0	8	
	bAVSD	25	0	25	
	uAVSD	3	3	0	
	Right ventricular hypoplasia	2	2	0	
Concomitant procedures (n)					
	PDA ligation	24	4	20	
	Atrioventricular valve repair	3	3	0	
	Aortic arch repair	8	0	8	
Associated co-morbidities (n)					
	Genetic abnormalities	11	1 (1.2)	10 (7.5)	0.054
	Preoperative respiratory support	3	1 (1.2)	3 (2.2)	1.000
	Lung/airway disease	93	30 (35.3)	63 (47.0)	0.094
Preoperative AVVR (n, %)					0.660
	Trivial or mild	163	66 (77.6)	97 (72.4)	
	Moderate	28	9 (10.6)	19 (14.2)	
	Severe	28	10 (11.8)	18 (13.4)	

mPAP, mean pulmonary artery pressure; ${\mathrm{SpO}}_{2}$, percutaneous arterial oxygen saturation; SV, single ventricle; TA, tricuspid atresia; DORV, double-outlet right ventricle; TGA, transposition of the great arteries; uAVSD, unbalanced atrioventricular septal defect; bAVSD, balanced atrioventricular septal defect; VSD, ventricular septal defect; COA, coarctation of the aorta; PDA, patent ductus arteriosus; AVVR, atrioventricular valve regurgitation.

### 3.3 Immediate Outcomes

A total of 10 patients died during their hospitalization, accounting for 4.6% of all patients; 6 patients died due to respiratory failure and 4 patients died due to sudden cardiac death. Among these cases, 40% of the patients had bAVSD, 20% had SV, 20% had multiple VSDs, 10% had DORV, 10% had TA, and 70% had moderate or severe AVVR. After the surgery, the mPAP decreased significantly from 38.0 (32.0–45.0) mmHg to 20.0 (15.0–25.0) mmHg (*p <* 0.05). There was no difference in postoperative mPAP between the univentricular repair group and the biventricular repair group. However, mortality was increased in patients with a higher postoperative mPAP. The postoperative outcomes are summarized in Table 2. Hospital mortality rate did not significantly differ between patients under 6 months and those over 6 months (5.4% *vs*. 4.0%, *p* = 0.622).

**Table 2.** **The postoperative outcomes of patients between univentricular repair group and biventricular repair group**.

Variable			Univentricular repair group (n = 85)	Biventricular repair group (n = 134)	*p* value
Postoperative mPAP (mmHg)		20.0 (15.0–25.0)	20.0 (15.0–25.0)	20.0 (15.0–24.0)	0.843
Postoperative ${\mathrm{SpO}}_{2}$ (%)		96.0 (90.0–100.0)	94.0 (90.0–100.0)	100.0 (97.0–100.0)	0.000
Ventilator use (h)		18.0 (8.0–34.0)	18.0 (8.0–35.5)	20.0 (8.0–31.0)	0.655
Duration of ICU stay (h)		48.0 (24.0–96.0)	48.0 (24.0–96.0)	48.0 (24.0–96.0)	0.759
Postoperative hospitalization stay (days)		7.0 (5.0–10.0)	6.0 (5.0–10.0)	6.0 (5.0–10.0)	0.890
Predischarge PAB velocity (cm/s)		423 (370–471)	413.0 (378–466.5)	425.0 (365.0–475)	0.696
Postoperative AVVR (n, %)					0.439
	Trivial or mild	181	68 (82.9)	113 (84.3)	
	Moderate	17	5 (6.1)	12 (9.0)	
	Severe	18	9 (11.0)	9 (6.7)	
Associated co-morbidities					
	Re-intubation	10	2 (2.4)	8 (6.0)	0.211
	Re-banding	1	1 (1.2)	0 (0.0)	0.208
	Pericardial effusion	11	4 (4.7)	7 (5.2)	0.864
	Atelectasis	19	3 (3.5)	16 (11.9)	0.031
In hospital motality (n, %)		10 (4.6)	3 (3.5)	7 (5.2)	0.744
Age at second-stage surgery (month)		43.0 (25.0–70.0)	47.0 (29.0–71.0)	33.0 (22.3–53.4)	0.286
Time interval for second-stage operation (month)		26.5 (16.0–48.0)	26.0 (14.0–42.0)	25.0 (16.5–44.0)	0.116

mPAP, mean pulmonary artery pressure; ${\mathrm{SpO}}_{2}$, percutaneous arterial oxygen saturation; ICU, intensive care unit; PAB, pulmonary artery banding; AVVR, atrioventricular valve regurgitation.

The preoperative mean grade of AVVR was 1.38 ± 0.70, which decreased to 1.25 ± 0.60 on predischarge echocardiography (*p* = 0.000). When comparing pre and postoperative echocardiography,14.6% (32) had a decrease in AVVR, 4.1% (9) had an increase in AVVR, and 81.3% (178) had no change in AVVR. Half of the patients with severe valve regurgitation before the PAB procedure did not show any improvement after the procedure. One patient required a repeated PAB after the initial PAB, and one patient underwent a pericardial window.

Genetic abnormalities, preoperative respiratory support, postoperative moderate or severe AVVR, and re-intubation were more common in the mortality group (*p <* 0.05). The significant factors associated with mortality were postoperative moderate or severe AVVR, re-intubation, and predischarge PAB velocity >388.5 cm/s after PAB (Table 3).

**Table 3.** **Univariable and multivariable logistic analyses of risk factors for in-hospital mortality after PAB**.

	Univariable analysis		Multivariable analysis	
	OR (95% CI)	*p *value	OR (95% CI)	*p* value
Male	0.204 (0.042–0.985)	0.048	–	-
Weight (kg)	0.775 (0.555–1.082)	0.134	–	-
Genetic abnormalities	0.556 (1.028–30.027)	0.046	–	-
Preoperative respiratory support	7.630 (0.721–80.768)	0.091	–	-
Postoperative AVVR (moderate or severe)	7.619 (1.898–30.585)	0.004	16.525 (2.586–105.608)	0.003
Postoperative mPAP (mmHg)	1.045 (0.977–1.118)	0.196	–	-
Postoperative ${\mathrm{SpO}}_{2}$ (%)	0.900 (0.818–0.990)	0.030	–	-
Ventilator use (h)	1.007 (1.003–1.001)	0.001	–	-
Duration of ICU stay (h)	1.005 (1.002–1.008)	0.001	–	-
Predischarge PAB velocity (cm/s)	0.998 (0.980–0.997)	0.009	0.988 (0.978–0.998)	0.001
Re-intubation	40.800 (8.885–187.364)	0.000	45.645 (4.996–416.818)	0.001
Pericardial effusion	5.556 (1.028–30.027)	0.046	–	-
Atelectasis	8.622 (2.191–33.927)	0.002	–	-

PAB, pulmonary artery banding; AVVR, atrioventricular valve regurgitation; mPAP, mean pulmonary artery pressure; ${\mathrm{SpO}}_{2}$, percutaneous arterial oxygen saturation; ICU, intensive care unit; OR, odds ratio; 95% CI, 95% confidence interval.

### 3.4 Follow-up Outcomes

**Fig. 1.**
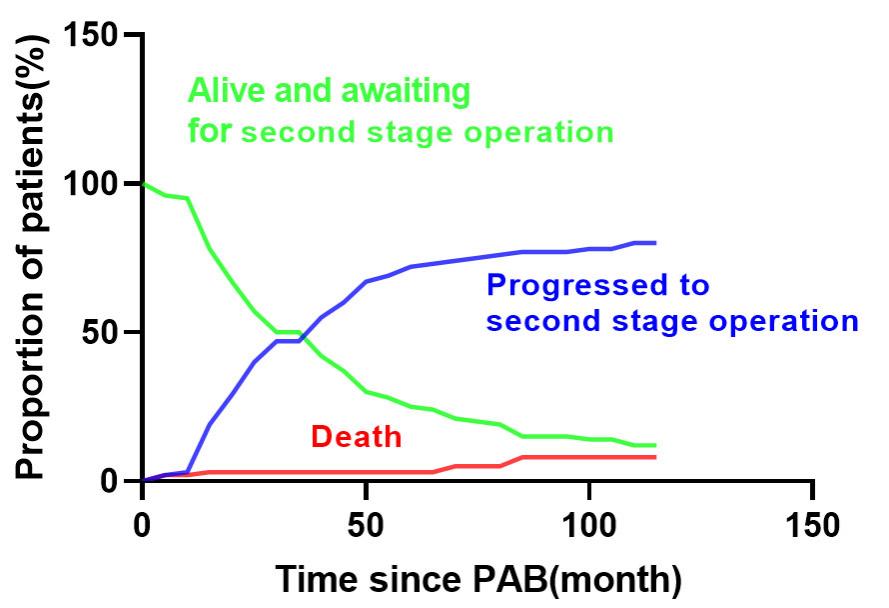
**Competing risk analysis of events after PAB.** Competing risk model showed that at the 10-year mark following the initial operation, 7.9% of patients had died, 79.8% had undergone the second-stage repair, while 18.6% remained alive and awaiting the second-stage operation. PAB, pulmonary artery banding.

The median follow up complete in 92.8% of the 186 survivors and averaged 33.0 (17.0–61.0) months. 147 (79.0%) patients underwent a second-stage operation. Survival rates were 96.9 ± 2.5% at 60 months and 92.1 ± 6.9% at 120 months post-PAB. The results of the competing risks analysis revealed that at the 10-year mark following the initial operation, 7.9% of patients had died, 79.8% had undergone the second-stage repair, while 18.6% remained alive and awaiting the second-stage operation (Fig. 1).

There was a noticeable improvement in the weight of all patients during the initial period following PAB, Before PAB, 63.5% of patients were below or at the 3rd centile for weight, while only 6.0% were above the 50th weight centile. At the time of the second-stage operation, 30% of patients remained at or below the 3rd weight centile, and 20% had reached the 25th weight centile.

During the follow-up, all patients had the PA band correctly positioned, and there was no evidence of band migration on echocardiography. There was no increase in the amount of AVVR or any ventricular systolic dysfunction observed on echocardiography in any patient.

**Fig. 2.**
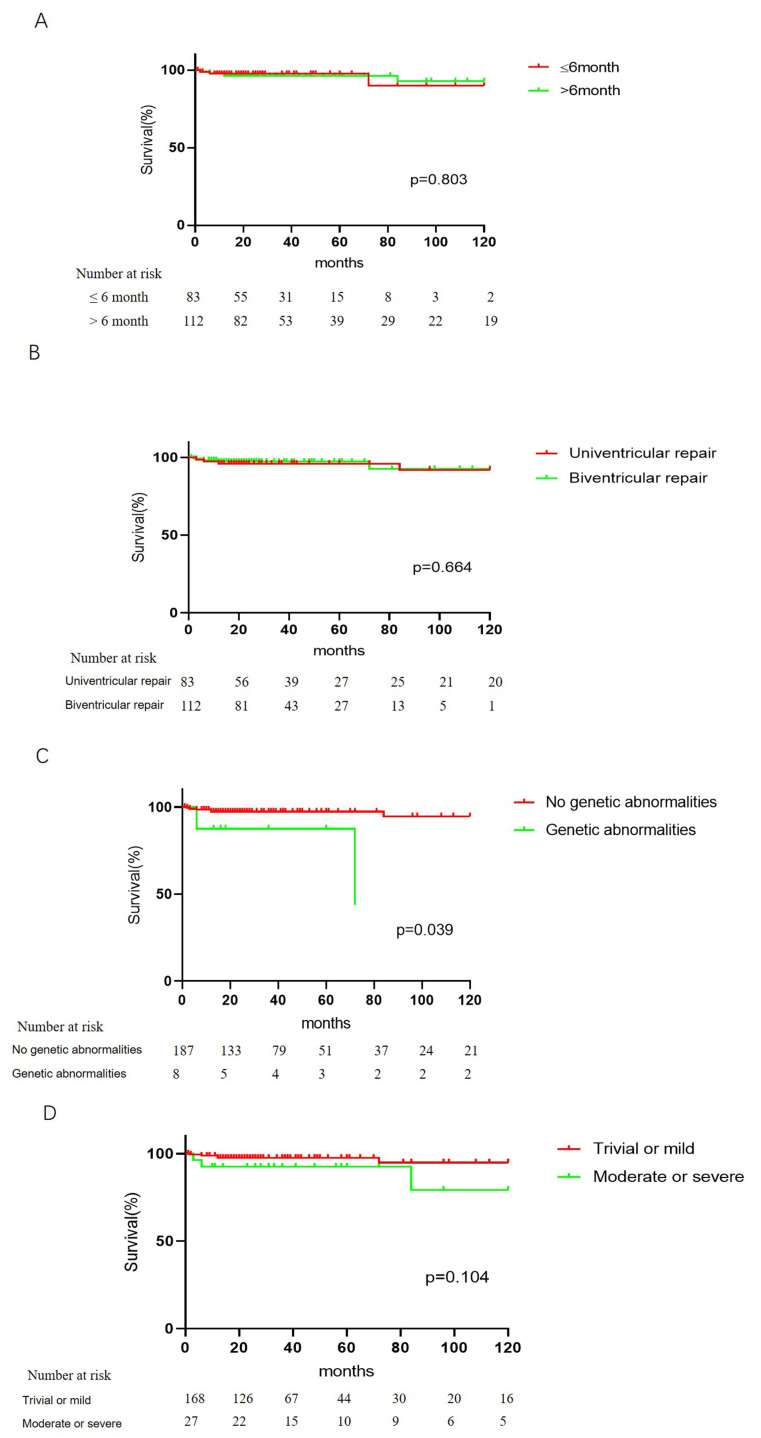
**Time-dependent survival after PAB in patients stratified by (A) different ages, (B) surgical approach, (C) postoperative AVVR and (D) genetic abnormalities.** PAB, pulmonary artery banding; AVVR, atrioventricular valve regurgitation.

There were no significant differences in long-term survival based on patient age, surgical approach, or postoperative AVVR. However, there were significant differences in long-term survival for patients with genetic abnormalities (Fig. 2).

**Fig. 3.**
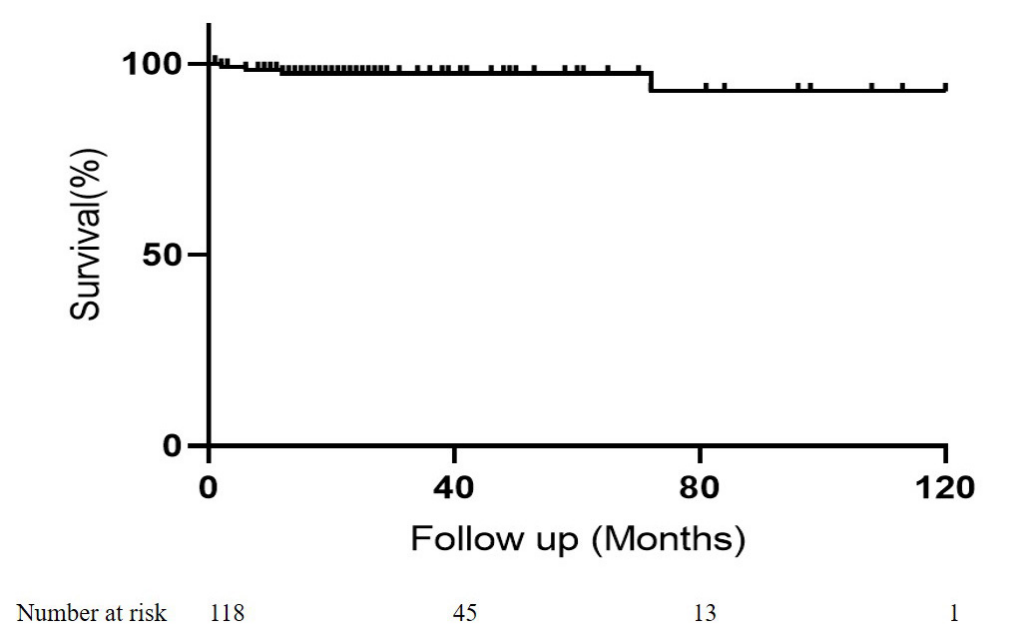
**Kaplan–Meier curves for overall survival biventricular repair group**.

After the initial PAB, 96 patients (71.6%) underwent a biventricular repair. 4 patients died before the repair. 2 patients with DORV and 1 patient with bAVSD, who passed away due to respiratory failure. 1 patient with DORV, who suffered sudden cardiac death. The inter-stage mortality was 3.4%. The survival rates were 97.4 ± 2.9% at 60 months and 92.7 ± 9.2% at 120 months post-PAB (Fig. 3).

**Fig. 4.**
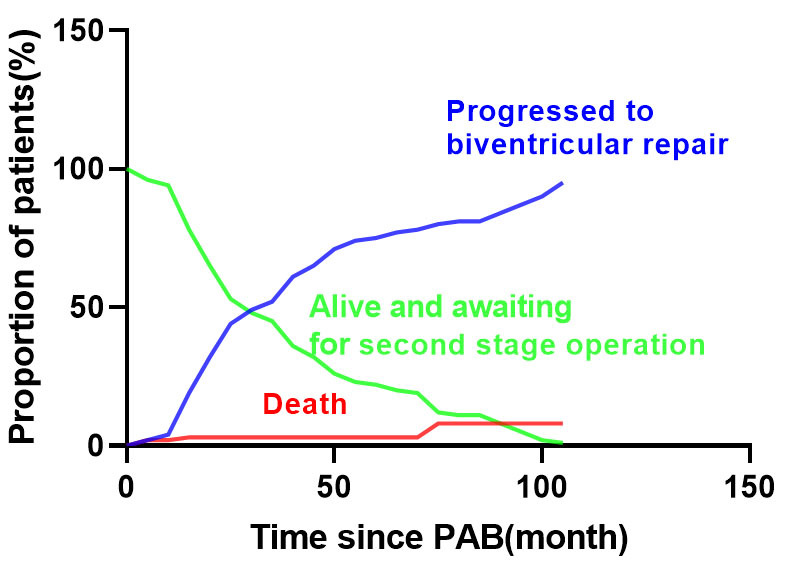
**Competing risk model showed that at the 10-year mark following the initial operation, 7.3% of patients had died, 95.1% had undergone the biventricular repair, while 4.5% remained alive and awaiting the biventricular repair.** PAB, pulmonary artery banding.

The competing risks analysis revealed that at the 10-year mark following the initial operation, 7.3% of patients had died, 95.1% had undergone the biventricular repair, while 4.5% remained alive and awaiting the biventricular repair (Fig. 4).

Among patients who achieved biventricular repair, the median duration was 28.0 months (ranging from 17.0 to 54.3 months), and the median age at the time of repair was 42.0 months (ranging from 23.4 to 68.2 months).

The biventricular repair procedures included 26 repairs for atrioventricular septal defects (AVSD), 39 VSD closures, 31 repairs for DORV, and complete de-banding. The post-repair mortality rate was 1 out of 96 patients (1.0%), and involved a patient who had been diagnosed with DORV and died due to acute left heart failure. None of the patients developed atrioventricular block.

**Fig. 5.**
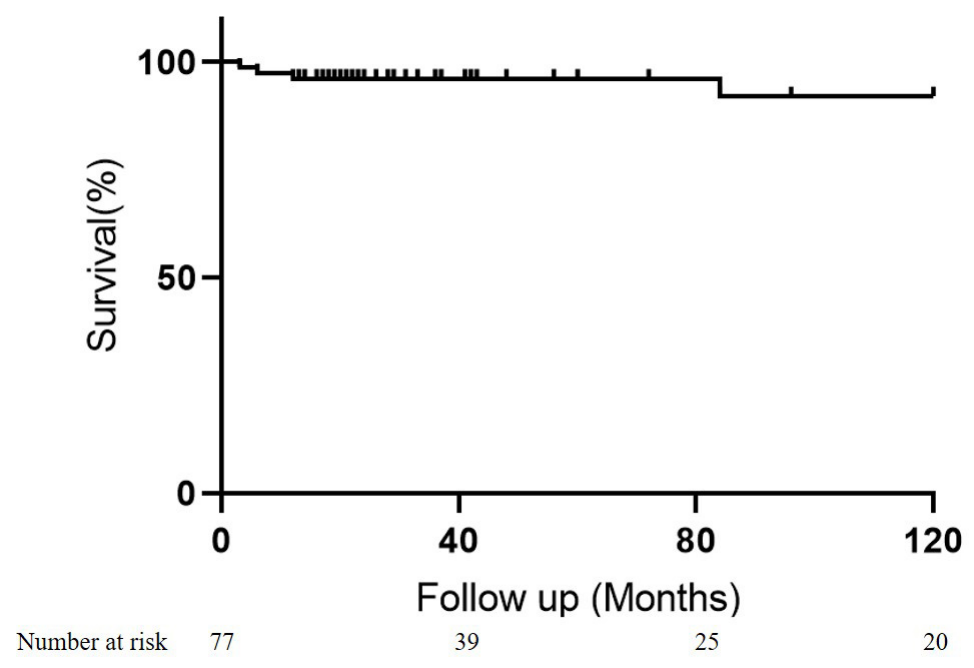
**Kaplan–Meier curves for overall survival univentricular repair group**.

After the initial PAB, 51 patients (66.2%) underwent the Glenn operation. 4 patients died before the Glenn operation. 1 patient with TA and 1 patient with DORV, passed away due to respiratory failure. 1 patient with SV suffered sudden cardiac death and in 1 patient with SV, the exact cause of death was unknown. The inter-stage mortality was 5.2%, and the survival rates were 96.1 ± 4.3% at 60 months and 92.1 ± 8.8% at 120 months post-PAB (Fig. 5).

**Fig. 6.**
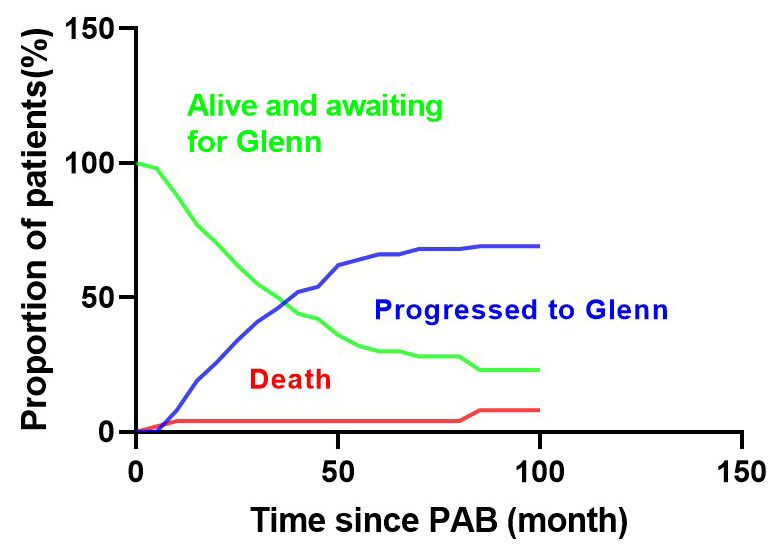
**Competing risk model showed that the competing risks analysis revealed that at the 10-year mark following the initial operation, 7.9% of patients had died, 68.9% had undergone the Glenn operation, while 28.6% remained alive and awaiting the Glenn operation.** PAB, pulmonary artery banding.

The competing risks analysis revealed that at the 10-year mark following the initial operation, 7.9% of patients had died, 68.9% had undergone the Glenn operation, while 28.6% remained alive and awaiting the Glenn operation (Fig. 6).

The median duration for patients who had the Glenn operation was 26.0 months (range: 14.0–42.0), and the median age at the time of repair was 47.0 months (range: 29.0–71.0).

**Fig. 7.**
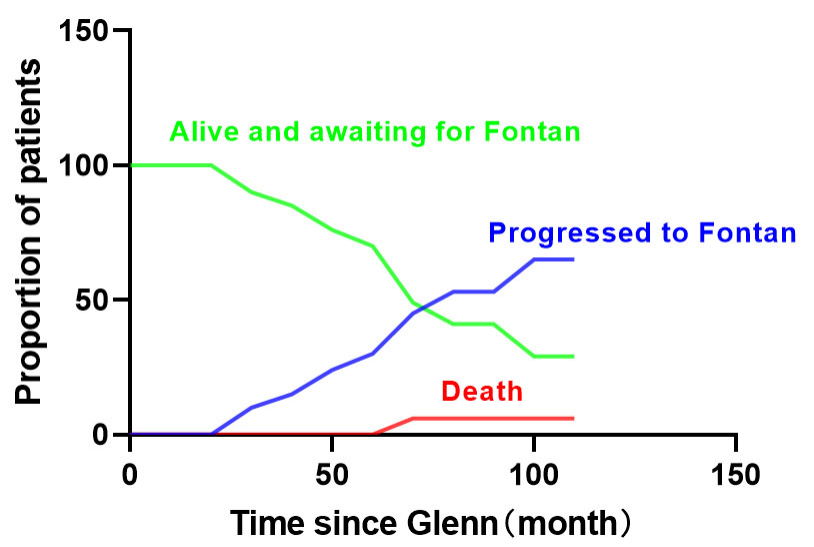
**Competing risk model showed that at the 8-year mark following the initial operation, 5.9% of patients had died, 64.5% had undergone the Fontan operation, while 33.4% remained alive and awaiting the Fontan operation**.

Out of the 51 Glenn operations, 21 patients (41.2%) proceeded to the Fontan operation, while 1 patient (1.9%) died prior to the procedure. The competing risk model demonstrated that the number of patients undergoing the Fontan operation started to increase at 20 months and reached its maximum at 80 months after the Glenn operation. The competing risks analysis revealed that at the 8-year mark following the initial operation, 5.9% of patients had died, 64.5% had undergone the Fontan operation, while 33.4% remained alive and awaiting the Fontan operation (Fig. 7).

The median duration for patients who achieved the Fontan operation was 51.0 months (range: 42.0–66.0), and the median age at the time of repair was 96.0 months (range: 81.0–130.9). There were no mortalities after the Fontan operation.

The patients were divided into two groups based on the age at which they underwent PAB. Group I consisted of 93 patients who were 6 months or younger, while Group II included 126 patients who were older than 6 months. There were no significant differences in preoperative and postoperative mPAP between the groups (*p* > 0.05). Furthermore, there were no differences in hospital mortality and inter-stage mortality after PAB. In Group I, 72.0% (67 patients) were able to undergo a second stage operation, with 81.7% (77 patients) receiving a biventricular repair. In contrast, in Group II, only 64.3% (81 patients) were able to undergo a second stage operation.

## 4. Comment

The first procedure performed in patients with congenital heart disease complicated with pulmonary hypertension is pulmonary artery banding. We believe that this procedure is beneficial in certain clinical scenerios, such as deferring corrective surgery for small and sick babies with biventricular circulation and increased pulmonary blood flow, as well as preparing patients with a univentricular heart and increased pulmonary blood flow for the Glenn and Fontan operations. The purpose of this study was to assess the outcomes of the staged repair approach. We believe that PAB is still an important part of the management of these patients, and the findings of this analysis can be used for comparison with the outcomes of high-risk primary repairs, to provide information for counseling parents and caregivers.

We have shown that PAB was associated with a relatively low early mortality in patients with PH. Earlier studies reported a 25% mortality with PAB [10]. Dhannapuneni *et al*. [11] in a group of 20 patients with AVSD who underwent PAB between 2000 and 2009 reported a 50% rate of in-hospital mortality. In contrast, Nagashima *et al*. [12] demonstrated no early hospital mortality and an inter-stage mortality of 5.26% in 38 patients with a variety of lesions, that was similar to our results. The mortality rate has been significantly reduced due to advancements in surgical techniques, precise timing of PAB, and enhancements in perioperative care [13].

PAB is essential in order to maintain low pulmonary vascular resistance during early infancy. Regrettably, there were still numerous patients who have not undergone surgery at the ideal time. In our previous studies, we found that PAB could still achieve good surgical results in patients older than 6 months, with a surgical mortality rate of 2.8% [14].

Our team believes that utilizing a more aggressive approach to target PH, such as implementing “triple” therapy that includes parenteral prostanoids and other agents, can be advantageous in reducing pulmonary artery pressure following surgery and preventing permanent remodeling of the pulmonary vasculature and pulmonary hypertension. Previous results from developing countries were not satisfactory, with a late mortality rate of up to 21% [15]. Results of pulmonary artery banding for atrioventricular septal defect over the past 30 years were still associated with a 18.6% inter-stage mortality in developing countries [16]. As a result, we have implemented a protocol during the inter-stage period, which includes regular administration of targeted pulmonary hypertension (PH) drugs and cardiotonic therapy, resulting in a lower inter-stage mortality rate.

In addition to mortality, the duration of ventilator use and hospital stay were also important indicators of treatment efficacy [17]. Our study found that the recovery after pulmonary artery banding was favorable. The patients required mechanical ventilation for an average of 18 hours, with a median hospital stay of 7 days. In comparison, a study by Afifi *et al*. [18], showed longer hospital stays and a greater need for mechanical ventilation in 125 patients who underwent banding at a median age of 41 (2–294) days. These findings suggest a significant difference between the two sets of data, potentially due to the inclusion of fewer younger patients in our study. However, good perioperative outcomes can also be achieved with PAB in older patients. Additionally, PAB can optimize patients for a safer cardiopulmonary bypass procedure during the definitive surgery. PAB has been successful in promoting weight gain before the second stage operation. Similar weight improvement was observed in all patients during the initial period following PAB, which aligns with the findings reported by Brooks *et al*. [19].

Previous studies have demonstrated that AVVR is a leading cause of morbidity and mortality [20, 21]. Buratto *et al*. [22] found that infants with AVSD did not experience significant worsening of left atrio-ventricular valve regurgitation between the time before PAB and before biventricular repair. In our own study, analysis of baseline and predischarge echocardiograms revealed a notable improvement in the degree of atrio-ventricular valve regurgitation. However, this improvement was not significant in patients who had severe regurgitation before PAB. Atrioventricular valve regurgitation has been recognized as a risk factor for both early and late mortality following single ventricle palliation [23]. King *et al*. [24] revealed that patients with a common atrioventricular valve were at risk for Fontan failure, particularly if they had moderate valve regurgitation. In our study, we observed that the degree of AVVR after PAB was associated with increased mortality. We hypothesized that the increased ventricular volume load may be linked to AVVR after PAB. As a result, in recent years, we have recommended repairing the AVVR during PAB, especially in patients where the regurgitation is moderate or more severe. This recommendation is based on our positive experience with repairing common AVVR.

Another goal of our study was to determine if patients undergoing PAB could ultimately proceed to a successful second-stage operation. In our study, 67.1% (147/219) of the patients successfully converted to the second stage operation. We compared patients in the univentricular repair and biventricular repair groups and found no statistically significant difference. However, we found that more patients in the biventricular group were able to successfully complete the second stage operation. These patients also had a shorter time interval prior to the second stage procedure and were younger than those in the univentricular repair group. We hypothesized that this could be due to the younger age of the initial stage patients in the biventricular group. Additionally, a higher proportion of patients under 6 months of age completed the second stage operation.

We also found no significant difference in late mortality among different patient age groups. Our results were consistent with Mukherji *et al*. [25], who found that performing PAB in older patients could have definite benefits in terms of survival and quality of life. For patients who missed the optimal surgical opportunity, our study demonstrated that older patients might still be able to undergo PAB and a staged operation following accurate preoperative assessment and targeted PH therapy.

In the cohort of patients who underwent univentricular repair, 51 patients (66.2%) underwent the Glenn operation, while 21 patients (41.2%) underwent the Fontan operation. The inter-stage mortality rate was found to be higher in univentricular cases compared to biventricular repair cases (5.2% *vs*. 3.4%, *p* = 0.336). The challenges associated with decreased right ventricular function after the univentricular repair include low pulmonary vascular resistance, and additional complications such as total anomalous pulmonary venous connection (TAPVC), AVVR, PDA, and COA [26]. It is recommended that these associated malformations be corrected simultaneously during the first-step procedure. The use of a PAB is believed to impede the progression of pulmonary hypertension and enable correction of the SV, leading to improved long-term outcomes for patients compared to those who are left without surgical intervention. Even patients who may not be ideal candidates for SV palliation are expected to have better survival with PAB, especially in the early years following surgery [27].

The results of our study demonstrate a notable association between the highest velocity of blood flow through the narrowed pulmonary artery caused by PAB, and the likelihood of death during the operation and in the long-term after the procedure. When the levels of oxygen saturation in the blood are within the acceptable range, we usually choose to apply a more restrictive PAB. This method is thought to have benefits in reducing excessive strain on the heart chambers and the blood vessels in the lungs. As a result of implementing this approach, we have observed positive outcomes in the treatment of these patients.

### Limitations

Our study was limited by the retrospective nature of the review. It can be challenging to assess the outcomes due to the diverse patient population and multitude of factors that contribute to a lack of progress. To address these limitations, we believe that randomized clinical studies and multicenter studies are needed.

## 5. Conclusions

The utilization of PAB is a feasible strategy for individuals afflicted with congenital heart disease, particularly those with pulmonary hypertension. Generally, the prognosis and outcomes following subsequent procedures tend to be favorable, particularly for patients aged six months and above. It is our opinion that by implementing a more restrictive PAB technique, coupled with the appropriate intervention for AVVR, the overall results achieved from these procedures can be significantly improved.

## Data Availability

All data points generated or analyzed during this study are included in this article and there are no further underlying data necessary to reproduce the results.
